# Retinal microvascular alterations related to diabetes assessed by optical coherence tomography angiography: A cross-sectional analysis

**DOI:** 10.1097/MD.0000000000006427

**Published:** 2017-04-14

**Authors:** Julien Gozlan, Pierre Ingrand, Olivier Lichtwitz, Agathe Cazet-Supervielle, Léa Benoudis, Michele Boissonnot, Samy Hadjadj, Nicolas Leveziel

**Affiliations:** a Department of Ophthalmology, CHU Poitiers; b Public Health Department, University of Poitiers; c University of Poitiers, UFR de Médecine et Pharmacie; d Biostatistics, INSERM CIC 1402, Centre d’investigation clinique, University Hospital of Poitiers; e Diabetology Department, Poitiers – Coordinating Center of the DIAB2NEPHROGENE Study; f INSERM 1084, University of Poitiers, France.

**Keywords:** capillary occlusion, capillary plexus, diabetic retinopathy, ischemia, macula, OCT angiography, retinal Imaging

## Abstract

Fluorescein angiography has been so far the gold-standard test to assess diabetic macular ischemia (DMI), a cause of irreversible visual impairment in diabetic patients. The aim of this study was to investigate foveal avascular zone (FAZ) and perifoveal microcirculation changes in eyes with nonproliferative diabetic retinopathy (NPDR) using optical coherence tomography angiography (OCTA), a new and noninvasive vascular imaging technique.

Cross-sectional study including eyes of diabetic patients with NPDR.

All patients underwent medical history, best-corrected visual acuity (BCVA) measurement, slit-lamp and fundus examination, multicolor imaging, SD-OCT, and swept-source OCT. OCTA was performed in order to assess macular superficial and deep capillary plexus, and swept-source OCT was performed to evaluate the central choroidal thickness.

Fifty-eight eyes of 35 patients with a mean age of 61.8 years (±12.1) with mean HbA1C level of 7.6% (±1.5) were included in this study. Among them, 19 eyes had mild NPDR, 24 eyes had moderate NPDR, and 15 eyes had severe NPDR. There was a significant progression between NPDR stages for FAZ grade (*P* < 0.0001), surface (*P* = 0.0036) and perimeter (*P* = 0.0001), and for superficial capillary plexus nonperfusion index (NPI) (*P* = 0.0009). Moreover, a significant correlation was found between NPI and BCVA (*P* = 0.007).

OCT angiography is a useful noninvasive tool to explore early phases of diabetic retinopathy, which are not routinely explore with fluorescein angiography and not precisely enough with color photographs. NPI and foveal avascular zone parameters are correlated with glycated hemoglobin in patients with NPDR. If confirmed by further studies, these results could represent a mean to sensibilize diabetic patients to their disease.

## 1 Introduction

Diabetes mellitus is a widespread condition affecting 366 million people worldwide.^[1]^ Diabetic retinal disease is one of the leading causes of blindness in Western countries.^[2]^ Visual impairment in diabetes is usually related to 3 different mechanisms: diabetic macular edema, complications of retinal neovascularization (mainly vitreous hemorrhage and retinal detachment), and diabetic macular ischemia (DMI).

DMI has been reported to affect approximately 7% of patients with diabetic retinopathy.^[3]^ This pathological condition is characterized by enlargement of the physiological capillary-free zone located at the center of the macula, also known as the foveal avascular zone (FAZ), along with perifoveal capillary dropout.

Various methods have been used to assess FAZ and perifoveal microcirculation: in vitro techniques on enucleated eyes as well as in vivo techniques, with psychophysical methods, fundus photography, and fluorescein angiography.^[4–7]^

The latter remains the gold-standard test, as it mainly explores the superficial capillary plexus which contains the perifoveal anastomotic capillary arcade defining the limits of the FAZ.^[8–10]^

Optical coherence tomography angiography (OCTA) is a newly available retinal vascular imaging technique, which is able to separately visualize superficial and deep macular capillary plexus.^[11]^

The aim of this study was to investigate foveal avascular zone and perifoveal microcirculation changes relative to mild, moderate, and severe nonproliferative diabetic retinopathy (NPDR) using the OCTA.

## 2 Methods

### 2.1 Experimental design

This cross-sectional study included diabetic patients affected with NPDR with appointments in the Department of Ophthalmology during July 2015. Patients were screened after informed consent was obtained. This study was performed according to the Declaration of Helsinki and was approved by the ethics committee of the French Society of Ophthalmology (institutional review board 00008855 Société Française d’Ophtalmologie).

### 2.2 Inclusion and exclusion criteria

Inclusion criteria were age of 18 years or more and presence of an NPDR in the studied eye, as assessed on nonmydriatic fundus color photographs covering the Early Treatment Diabetic Retinopathy Study (ETDRS) 7-standard fields. For each patient, one or both eyes meeting those criteria were included.

Noninclusion criteria were: presence of media opacities preventing reliable retinal imaging and fundus examination, macular edema with retinal thickness preventing good visualization of the FAZ by OCTA, other retinal diseases (age-related macular degeneration, macular hole, epiretinal macular membrane, foveoschisis, and foveal hypoplasia), history of vitreoretinal surgery, and history of macular laser grid.

### 2.3 Data collection

All patients included in the study underwent comprehensive ophthalmologic examination including best-corrected visual acuity (BCVA) measurement on ETDRS scale, slit-lamp biomicroscopic and fundus examination, Spectral-Domain Optical Coherence Tomography (volume mode of 49 B-scans, centered on the fovea), macular 30 degree MultiColor retinography (Spectralis HRA + OCT; Heidelberg Engineering, Heidelberg, Germany) as well as ETDRS 35 degree 7-standard fields color retinal photographs (Topcon TRC; Topcon, Tokyo, Japan) for retinal assessment, Swept-Source Optical Coherence Tomography (DRI OCT-1 Atlantis; Topcon, Tokyo, Japan) for choroidal thickness assessment, and Optical Coherence Tomography Angiography (Optovue RTVue XR Avanti; Optovue Inc, Fremont, California) for macular retinal vascularization assessment.

Diabetic retinopathy was graded according to the International Diabetic Retinopathy Severity Scale, as previously described.^[12]^

Demographic data (age, gender), general medical history (smoking, high blood pressure, hyperlipidemia, cerebral vascular accident, myocardial infarction, and amputation), diabetic medical history (type of diabetes, disease duration, most recent level of glycated hemoglobin, oral diabetes medication, and insulin therapy), and ophthalmologic history (cataract surgery, intravitreal antivascular endothelium growth factor injections) were also collected.

### 2.4 Optical coherence tomography angiography

For each included eye, an OCTA of the superficial and deep capillary plexus was performed as previously described.^[11]^ This instrument uses a novel noninvasive and dyeless technique, which performs repeated sequential Optical Coherence Tomography cross-sectional scans at the same location on the retina in order to capture the dynamic motion of the erythrocytes. Thus, this device permits high-resolution imaging of the perfused vascular structure within specific layers of the retina with a 22-μm beam width. It has an A-scan rate of 70,000 scans per second, using a light source centered on 840 nm and a band-width of 45 nm. The tissue resolution is 5 μm axially, and the beam is 22-μm wide.

A 3 × 3 mm OCTA acquisition centered on the fovea was performed. The “en face” image was then automatically segmented with an inner boundary 3 μm beneath the internal limiting membrane and an outer boundary 15 μm beneath the inner plexiform layer (IPL) to obtain images of the superficial capillary plexus. The segmentation was carried out with an inner boundary 15 μm beneath the IPL and an outer boundary 70 μm beneath the IPL in order to obtain deep capillary plexus images.^[13]^

### 2.5 Superficial and deep capillary plexus image analysis

Each superficial capillary plexus image was analyzed using ImageJ 1.48 software (NIH, Bethesda, MD).^[13]^ We manually traced FAZ contours in order to assess its different parameters. After setting the image scale to 3 × 3 mm in the software parameters, we were able to automatically obtain measurement of the perimeter and area of the FAZ.

Similarly, the surface of the central nonperfused area visible on the deep capillary plexus image was measured.

**Figure 1:**
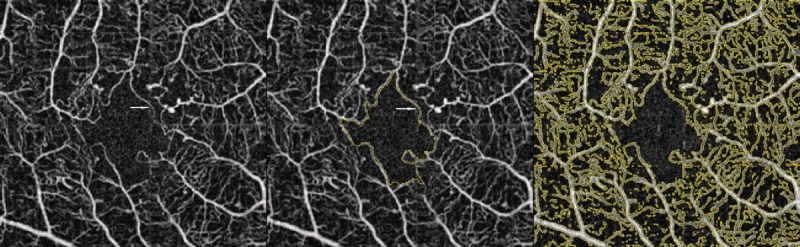
Optical coherence tomography angiography superficial capillary scan of a right eye with moderate nonproliferative diabetic maculopathy. (Left) The foveal avascular zone appears to be enlarged, with presence of perifoveal capillary dropout (arrow). (Center) Semiautomatic selection of the foveal avascular zone (arrow). (Right) Automatic selection of nonperfused areas.

Furthermore, a nonperfusion index (NPI) of the total superficial capillary plexus image area was assessed using the same software by calculating the ratio between the cumulative area of pixels of which the brightness was beneath the value of 45 and the total area of the picture (Fig. 1).

### 2.6 FAZ contour assessment

The extent of FAZ damage was also assessed using the ETDRS gradation system from 0 (normal FAZ) to 4 (FAZ contours completely altered), as previously described.^[14,15]^

### 2.7 Statistical analysis

Statistical analysis was performed with SAS software, version 9.3 (SAS Institute Inc, Cary NC). Qualitative variables are presented as number and percentage. Quantitative variables are presented as means and standard deviations. Nonparametric ANOVA comparing the 3 NPDR groups used the nonparametric Kruskal–Wallis test. Qualitative variables were compared by Fisher exact test. Univariate analysis of FAZ parameters was based on Spearman rank correlation coefficient when relating to quantitative variables and Mann–Whitney nonparametric test when relating to qualitative factors. Multivariate analysis used linear covariance analyses for each FAZ parameters. Covariates were entered in the analysis on a *P*-value less than 0.20 from the univariate analysis, then a backward elimination procedure retained only independently significant variables. A logarithmic transformation was performed in order to normalize variable distribution if necessary. *P* < 0.05 was considered statistically significant. In this exploratory analysis, no correction was applied to account for intraclass correlation between eyes belonging to the same patient.

## 3 Results

### 3.1 Demographic data and medical history

**Figure 2:**
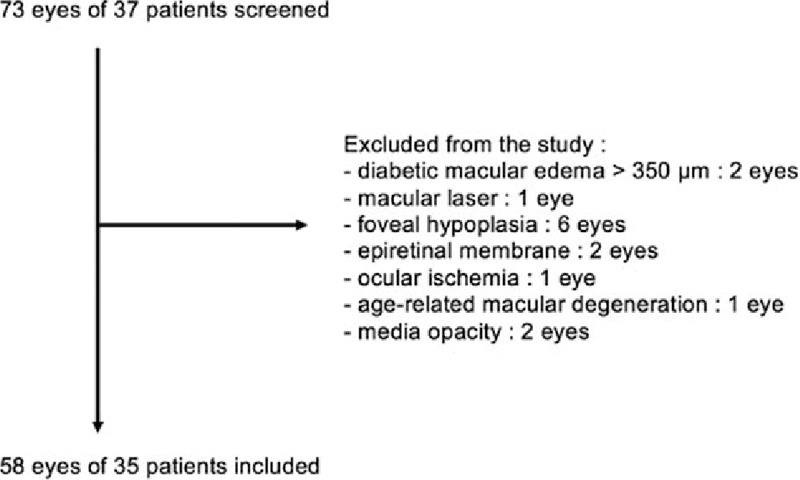
Flow chart of study patients.

Fifty-eight eyes of 35 patients (mean age = 61.8 ± 12.1 years) with NPDR were included in this study (Fig. 2).

Among those 35 patients, there were 22 men (63%) and 13 women (37%); type 2 diabetes affected 30 patients while 5 patients had type 1 diabetes. Mean diabetic duration was 18.3 ± 9.9 years, mean glycated hemoglobin A1C (HbA1C) level was 7.6 ± 1.5%, and 22 patients (63%) had oral medication and 29 patients (83%) had insulin therapy. Cardiovascular history of smoking, high blood pressure, and dyslipidemia were present in the majority of patients. These results are presented in Table 1.

**Table 1:**
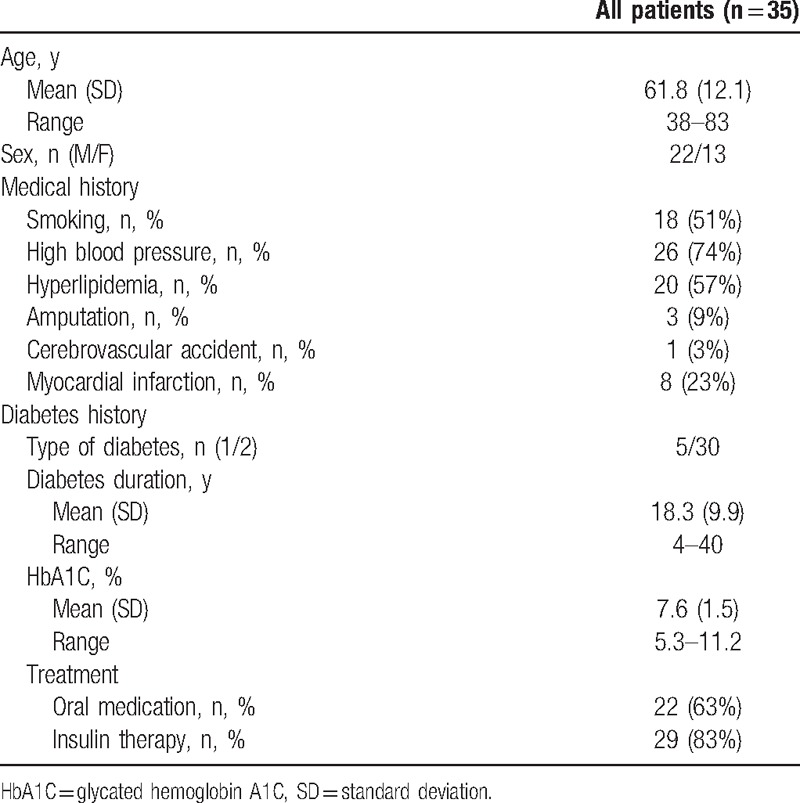
Patient demographic characteristics and medical history.

### 3.2 Ocular history, functional, and anatomical characteristics

Mean BCVA was 81.2 ± 12.3 letters (15–99). Only 1 eye had intravitreal antivascular endothelial growth factor history. Among the 58 included eyes, 19 eyes (32.8%) had mild NPDR, 24 eyes (41.4%) had moderate NPDR, and 15 eyes (25.8%) had severe NPDR (Table 2).

**Table 2:**
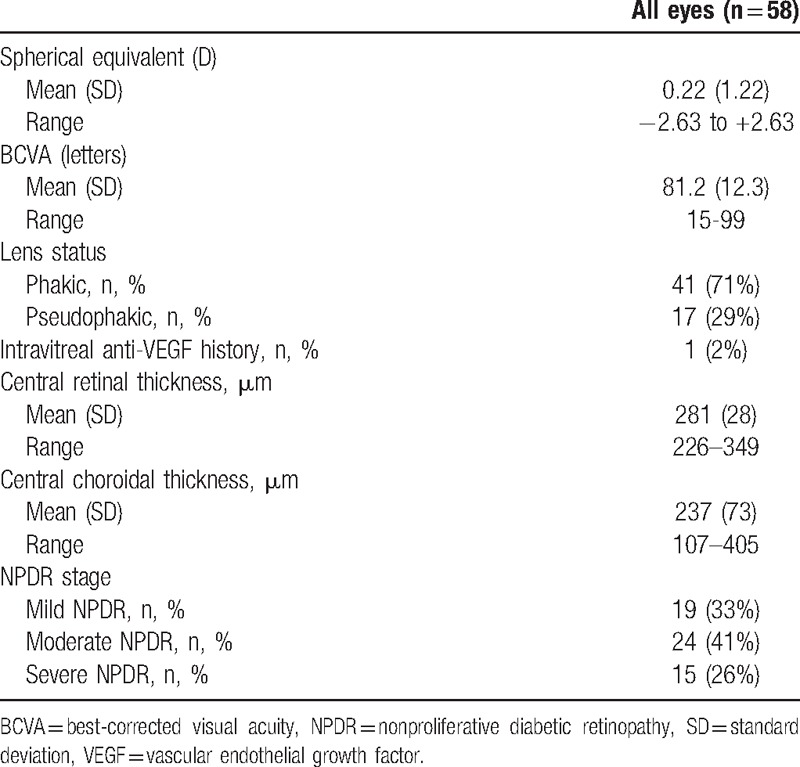
Ocular history, functional, and anatomical characteristics.

### 3.3 NPDR group comparison analysis

Table 3 summarizes the comparison analysis between the 3 NPDR groups. No significant differences for age, sex, medical history, diabetes history, and ocular characteristics were observed among the groups, except for spherical equivalent (*P* = 0.034), oral diabetic medication (*P* = 0.002), and HbA1C (*P* = 0.049).

**Table 3:**
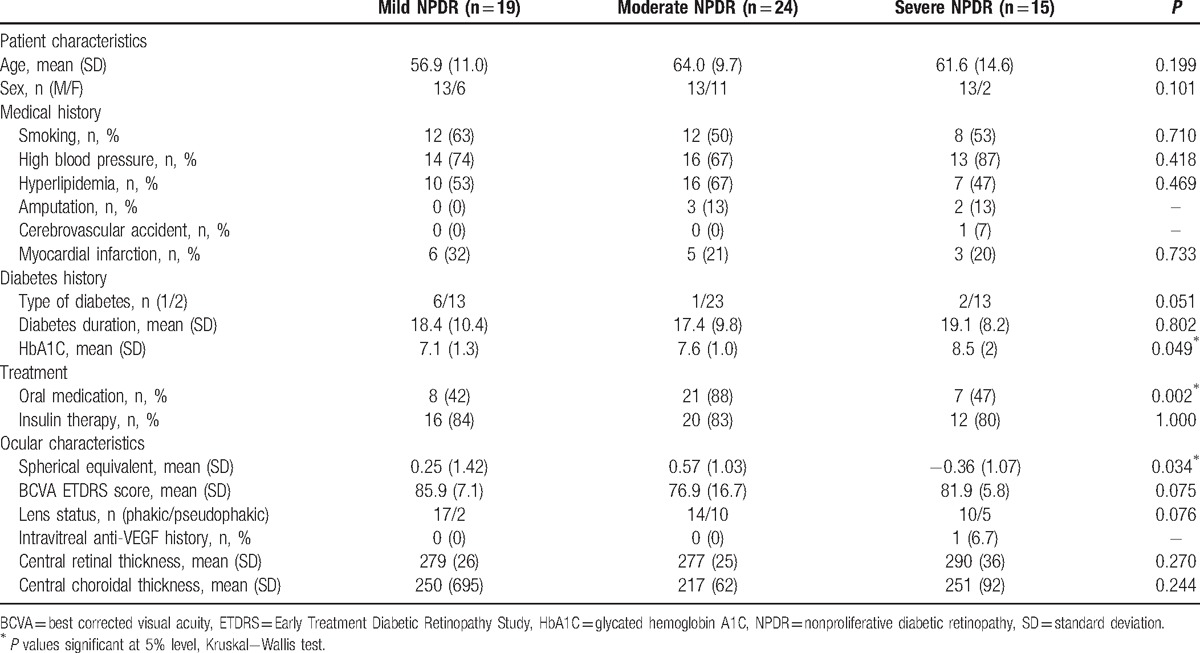
Comparison analysis between the 3 NPDR groups.

### 3.4 Relationship between NPDR status and OCTA data

**Figure 3:**
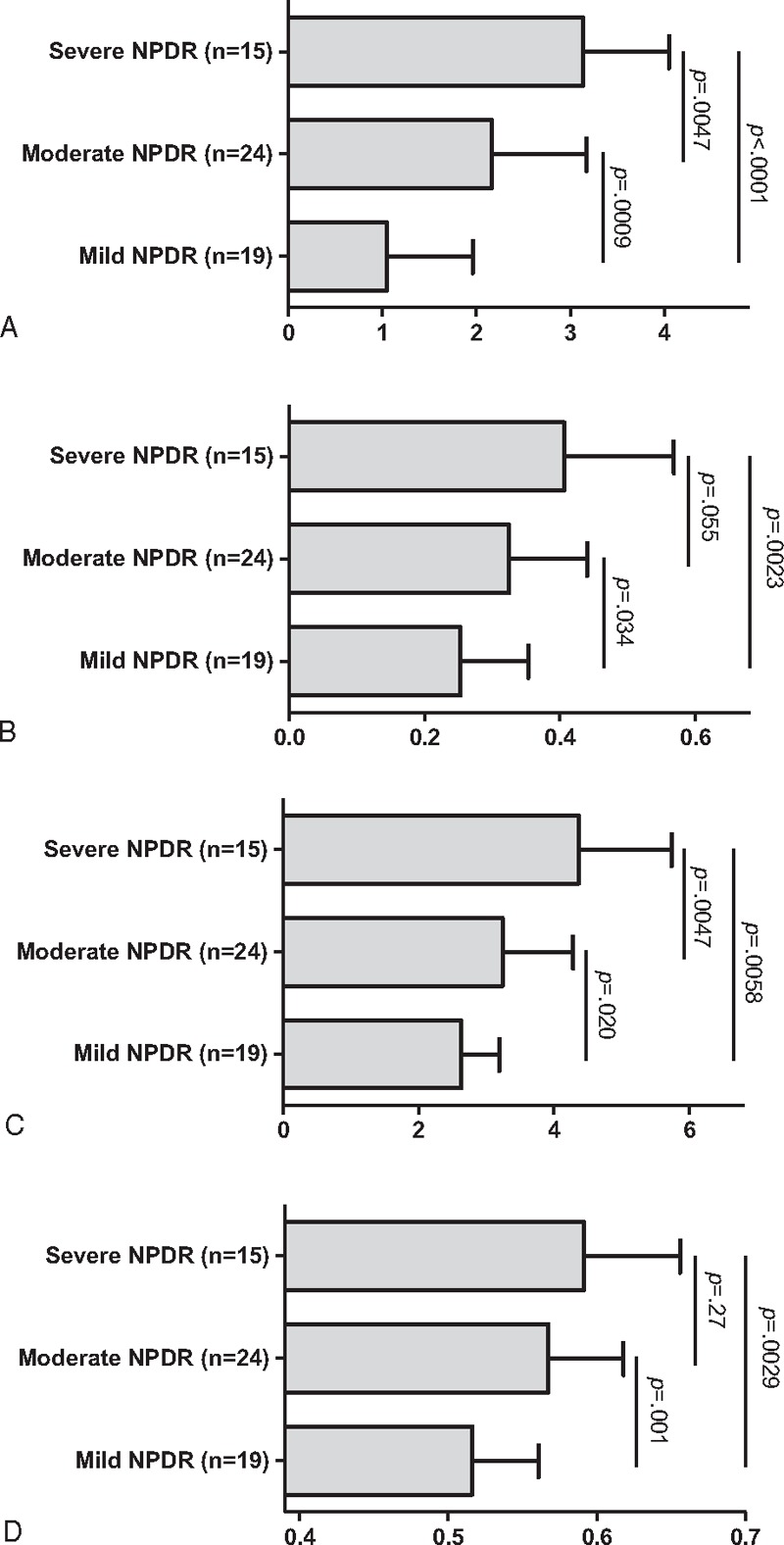
Comparison of OCTA parameters according to the NPDR status. (A) FAZ grade, (B) FAZ surface (mm^2^), (C) FAZ perimeter (mm), and (D) nonperfusion index. Results expressed as mean and standard deviation. Significance *P*-values from the Kruskal–Wallis test. FAZ = foveal avascular zone, NPDR = nonproliferative diabetic retinopathy, OCTA = optical coherence tomography angiography.

**Figure 4:**
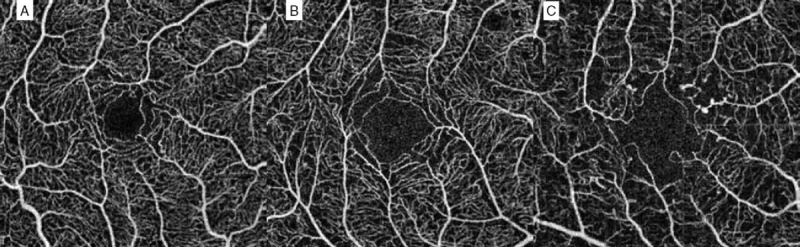
Optical coherence tomography angiography (OCTA) scans of the three different groups of nonproliferative diabetic retinopathy. (A) mild NPDR; (B) moderate NPDR; (C) severe NPDR.

There was a statistical difference between the 3 NPDR groups for FAZ grade (*P* < 0.0001), FAZ surface (*P* = 0.0036), FAZ perimeter (*P* = 0.0001), superficial capillary plexus (SCP) NPI (*P* = 0.0009), and deep capillary plexus (DCP) central nonperfusion area surface (*P* = 0.0002). These results are reported in Fig. 3. Moreover, OCTA scans of the 3 different groups of NPDR are presented in Fig. 4.

### 3.5 Analyses of FAZ parameters

Univariate analysis found significant associations between pseudophakia and FAZ surface (*P* = 0.033), between HbA1C and FAZ grade (*P* < 0.0001), surface (*P* = 0.0006), and perimeter (*P* = 0.0136). NPI was related to amputation history (*P* = 0.020), diabetes duration (*P* = 0.014), HbA1C (*P* = 0.039), and BCVA (*P* = 0.019). These results are indicated in Table 4.

**Table 4:**
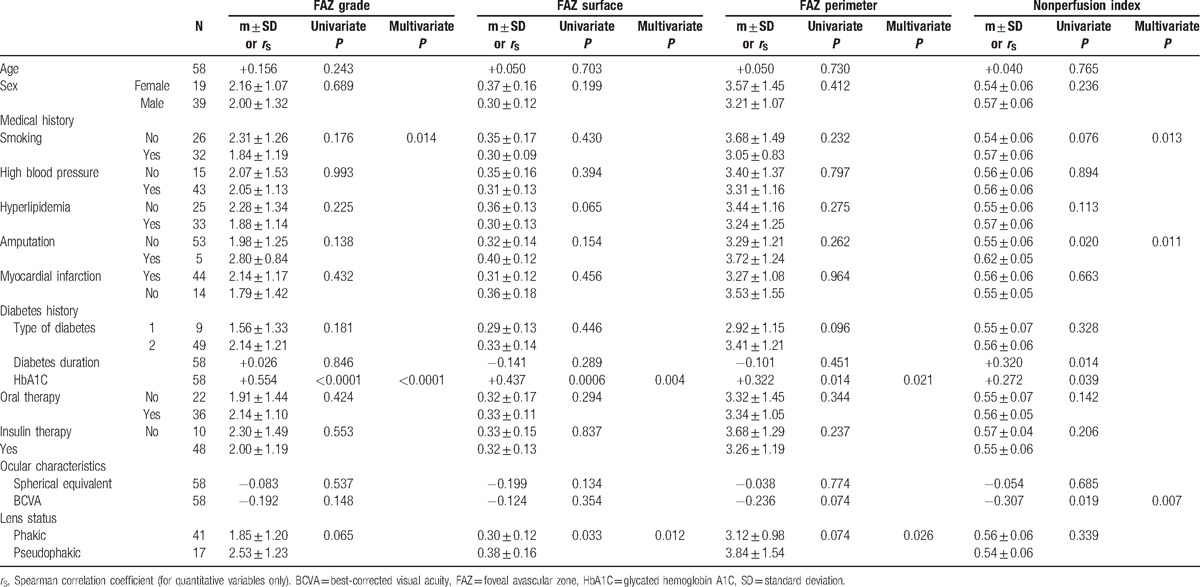
Univariate and multivariate analysis of FAZ parameters and nonperfusion index.

Multivariate analysis retained a positive correlation was observed between HbA1C and FAZ grade, surface, and perimeter. A negative correlation was found between BCVA and NPI. Smoking was also associated with FAZ grade and NPI. These results are reported in Table 4.

## 4 Discussion

Over the past decades, FA has been the gold standard procedure to explore DMI. However, it has limitations, the most common being fluorescein injection, which can trigger frequent benign symptoms such as nausea and, rarely, severe reactions such as anaphylactic shock.^[16]^ Moreover, there are specific drawbacks in this indication, as FA quality may not always be good enough for reliable analysis of FAZ, and retinal vascularization may be difficult to analyze in case of retinal pigment epithelium atrophy.^[17]^ Furthermore, given its potential complications, FA is not recommended during the initial stages of diabetic retinopathy. In this study, we performed detailed quantitative analysis of FAZ and perifoveal microcirculation to assess DMI in patients with NPDR using OCTA, a novel noninvasive technique that facilitates imaging of perfused retinal and choroidal vascular structures within specific layers. Recent literature shows that OCTA has a wide range of potential applications in ophthalmology, including glaucoma and chorioretinal disorders such as exudative age-related macular degeneration, macular telangiectasia type 2, and diabetic retinopathy.^[18–20]^

DMI assessment has a major impact on visual function, leading to loss of retinal sensitivity, loss of contrast sensitivity, electroretinographic changes, and lower visual acuity.^[21–23]^ Histopathologically, DMI has been shown to induce inner retinal changes, with loss of ganglion cells, attenuation of the inner nuclear layer, and hyalinization of the retinal capillary network.^[24]^

In this study, retinal macular microvascularization evaluated by OCTA differed significantly according to the stage of NPDR. This is, to our knowledge, the first study investigating FAZ and perifoveal microcirculation in NPDR patients, using a noninvasive device available for daily clinical practice.

A statistical difference for each FAZ parameter (grade, surface, and perimeter) was observed between the NPDR groups, with a progressive increase of parameters according to NPDR stage. These results are not surprizing, as a significant increase in FAZ size is a well-known FA feature of diabetic retinopathy.^[25–28]^ Thus, OCTA is able to provide quantitative analysis of FAZ, and the results are equivalent to those achieved with FA.

Deep capillary plexus central nonperfused area was also significantly different between the 3 NPDR groups. However, OCTA image segmentation of the deep capillary plexus on OCT B-scan seemed relatively random and unreliable, and its clinical significance needs to be clarified.

Finally, a statistical difference for NPI between the 3 NPDR groups was observed, with a progressive increase with NPDR stage. This is, to our knowledge, the first study to evaluate thoroughly and quantitatively macular microvascularization within a 3 × 3 mm square centered on the fovea in diabetic patients using an automated tool. Previous studies mostly used, besides FAZ measurements, a perifoveal intercapillary area index requiring manual marking of intercapillary spaces surrounding the fovea, with a different measuring protocol from one study to another. In addition to being automated, NPI has the advantage of taking into account, in a single standardized measurement, both FAZ surface enlargement and perifoveal capillary dropout.

Moreover, unlike FAZ parameters, in this study NPI was significantly associated with BCVA. In the literature, the correlation between BCVA and FAZ surface has not been consistently established. Although it was observed in a small series of 15 patients conducted by Arend et al,^[29]^ it did not appear in a more recent retrospective study conducted by Sim et al^[30]^ on FA images of a large cohort of patients, who nevertheless found a strong significant and independent association between papillomacular ischemia and BCVA. Thus, in our study, correlation between BCVA and NPI represents a new argument underlining the importance of perifoveal circulation in the occurrence of visual impairment secondary to DMI.

In this study, HbA1C was positively correlated with FAZ surface, FAZ perimeter, FAZ grade, and NPI. In previous studies, however, associations between HbA1C and macular microcirculation parameters have been inconsistently described. In patients either without or with background retinopathy, Sander et al^[31]^ found a significant correlation between HbA1C and perifoveal intercapillary area, but not with FAZ surface. Conrath et al^[14]^ likewise failed to find evidence of a correlation between FAZ indexes (surface, grade) and HbA1C.

Another positive correlation was observed between smoking status and FAZ grade and NPI, which is to our knowledge the first report of this association. In the context of diabetes, HbA1C and smoking correlated to the progression of FAZ and NPI indexes in NPDR patients could represent a mean to sensibilize this population.

We acknowledge some limitations to this study. Indeed, OCTA was not compared to FA, the gold standard for FAZ evaluation. However, in NPDR assessment this comparison could be considered as questionable on account of ethical considerations. The sample size of this study was relatively small, but it was an exploratory analysis and sample size was determined by the statistician (PI).

In conclusion, this study shows that OCTA is able to explore both superficial and deep macular capillary plexus in NPDR. With this noninvasive approach, progressive FAZ grade worsening and FAZ surface enlargement were observed during progression of NPDR. In addition, NPI, a newly described index, allows DMI assessment in a single automated and protocolized measurement taking into account both foveal and perifoveal microvascularization. Finally, even at early stages of diabetic retinopathy, HbA1C and smocking status seem to be associated with FAZ surface enlargement and NPI progression. If confirmed, these results could have therapeutic implications.

## Acknowledgments

The authors thank Jeffrey ARSHAM, an American medical translator, for rereading and reviewing our original English-language text. We are indebt to Optovue RTVue XR Avanti for providing us a device during the study.
